# Hospital and Health News

**Published:** 1924-02

**Authors:** 


### HOSPITAL AND HEALTH NEWS

### New Infirmary for Harrogate.

The committee of the Harrogate Infirmary has decided to purchase the Grove House Estate for £8,000, and there to build a new infirmary.

### Next Hospital Sunday.

Hospital Sunday for 1924 has been fixed to take place on June 22, when it is hoped that the collection will recover the loss that was shown last year over the 1922 total.

### A Presentation.

Mr. H. J. Packe, who has been for so many years the secretary of the Torbay Hospital, has been presented on his retirement with a cheque for a hundred guineas from the hospital's friends.

### " Wellcome " Brand of Insulin.

The increased demand for insulin, resulting in more economic production, has enabled Messrs. Burroughs, Wellcome & Co. to supply it henceforward at the reduced price of 12s. 6d. per phial containing 100 units (10 doses) in 5 c.c.

### A Hospital Architect.

Mr. G. Washington Browne, the new president of the Royal Scottish Academy, is the architect who designed the Edinburgh Royal Infirmary for Sick Children, and is the first architect to be elected to the presidency.

### Accident to Mr. C. E. A. Bedwell.

Mr. C. E. A. Bedwell, House Governor of King's College Hospital, has, unfortunately, been a patient in the casualty department of the institution. When leaving a tramcar opposite the hospital entrance he broke his leg.

### Milkmen's Hospital Week.

It is remarkable that a Milkmen's Hospital Week in the Thames Valley raised more than twice as much as was collected on the local hospital day from which the plan originated, and a total of more than £1,500 was raised for the Richmond Royal Hospital.

### Lectures by Sir Robert Armstrong-Jones.

Sir Robert Armstrong-Jones is delivering four lectures on Physic on January 29 and the three following days at Gresham College. The lectures, to which admission is free, will begin at 6 p.m., and other subjects are Headache, Rheumatism, Gout and Obesity.

### Unit System at the London.

We are asked to correct a statement made in our January issue to the effect that the London Hospital had found the unit system unsatisfactory and was abandoning it. The Hospital has decided not to have a surgical unit for which there seemed no scope, but its medical unit is doing excellent work and will be continued.

### Care of the Teeth.

The British Dental Association have issued a useful and compact little pamphlet on " Decay of the Teeth " (3d.), together with a card containing " Three Things to Remember " and " Three Things to Do." It is suggested that the card should be hung where it can be seen while dressing. But, really, this is taking our teeth a little too seriously.

### Foreign Health Visitors at Bradford.

Health visitors from France, Hungary, Norway, Denmark, Finland and Latvia visited Bradford recently to study the city and health services. They stayed there for a fortnight with the Matron of the Bradford Royal Infirmary. The Health Visitors have come to England under the auspices of the League of Red Cross Societies and are taking an international course in public health at Bedford College.

### The Late Mr. Custance.

The death of Mr. H. N. Custance was a sad event in the jubilee year of the Metropolitan Hospital Sunday Fund. When it was formed in 1873, under the mayoralty of Sir Sydney Waterlow, Mr. Custance was the first permanent secretary, a post that he held until 1902, when he retired upon a pension. In that period the collection rose from £27,000 to more than double, and the cherished aim of £100,000 a year is becoming practical.

### Lowestoft and North Suffolk Hospital.

The new memorial Wards at the Lowestoft and North Suffolk Hospital, which provide sixty beds and six beds in private wards, were ready for occupation in the middle of January, and before long the X-Ray department will be ready. The total cost of building and furnishing has been £23,000, and £7,000 is the total deficit. The children in the welfare centres have followed those in the schools in contributing to the hospital. A new nurses' hostel is contemplated.

### Rockefeller Gift to Edinburgh University.

The Rockefeller Foundation Trustees have offered to set a capital sum of £50,000 at the disposal of Edinburgh University for the erection of a clinical laboratory in conjunction with the Royal Infirmary, and for the completion of the endowment of the Professorship of Surgery on a full-time basis. In addition the trustees have offered an annual grant of £1,750 for at least five years, from which certain salary charges in clinical medicine and clinical surgery would be met.

### "Ye Bowe Bells Magazine."

Messrs. Edward Cook, the well-known soap manufacturers, have for many years published a capital little house paper, *Ye Bowe Bells Magazine*, which is edited by Mr. H. T. Morley-Attwell. Written and illustrated by the staff without professional assistance of any kind, it has hitherto been, as the editor says, "a magazine produced by the family for family use." Now, however, owing to its ever-increasing circulation, it is widening its scope, will publish more frequently and tell its readers something of the work as well as of the playtime of this great firm. Once distributed "among our own people only, it is now sent to the four corners of the world and our mail list includes every civilised country."

### Royal Victoria Infirmary, Newcastle.

At the last quarterly court of the Governors of the Royal Victoria Infirmary, Newcastle-upon-Tyne, Lord Armstrong, chairman of the House Committee, who presided, said that, taken as a whole, the report was a satisfactory one, more especially on the financial side. That was the first occasion on which they had been able to attend a quarterly court with a balance in their favour. They expected that, at the end of the year, there would be a surplus income over expenditure of £2,000; but against that there would be still outstanding £2,300 on the orthopædic account.

### Northampton General Hospital.

Sir James Crockett has given £5,000 to the Northampton General Hospital towards the £15,000 scheme for the building of a new isolation block. This gift is the most recent of quite a series of donations to this institution, in the administration of which Sir James has for many years shown a strong personal interest and taken a prominent part. In 1915 the firm of Crockett and Jones gave £384 towards the equipment of one of the pavilions for the wounded soldiers. A year later Sir James Crockett made a present of £1,000 for the endowment of a bed in memory of his son, Lieut. C. J. Crockett, who died as the result of the war. Only two years ago he paid £200 towards the building of a new lecture-room for the nursing staff.

### Waterproof Sheeting.

An excellent waterproof bed sheeting is manufactured by the Provincial Waterproof Co., Ltd., of Provincial Works, Strangeways, Manchester. It is obtainable in red and drab, and is used in many institutions throughout the country. It is guaranteed to resist the action of ammonia, carbolic acid, turpentine, etc., and can easily be cleansed by using soap and water. This firm also manufactures hot water beds, pillows, bottles, red rubber air cushions, operation gloves, and every description of surgical india-rubber goods.

### Blind Masseurs.

All the blind candidates entered for the recent Massage Examination of the Chartered Society of Massage have successfully qualified. They were all trained in the Massage School of the National Institute for the Blind by an entirely blind staff. Blind men and women are prepared for massage examinations under exactly similar conditions to those for sighted candidates. Amongst the masseurs practising in London are a doctor's son, a B.A. and a chartered accountant. The majority of blind masseurs and masseuses have their own private clinics, equipped with modern apparatus, where patients can be received for treatment. The secretary of the Massage Department, National Institute for the Blind, 224, Great Portland Street, will be glad to answer any inquiries.

### Deaths of X-Ray Workers.

The death has occurred of Sir Archibald Douglas Reid, superintendent of the X-Ray Department at St. Thomas's. He was one of the leading authorities in radiology and the President of the War Office X-Ray's Committee. He had lost several fingers as the result of his work. He was about to go to Egypt to X-ray Tutankhamen in his tomb at Luxor. The death of Mr. Thomas John Oliver, for many years X-Ray operator at Leeds General Infirmary, has taken place after much suffering, the result of X-Ray dermatitis. His right hand first became affected, then his left arm, and an excision in his left elbow was necessary. In spite of this, he worked bravely until in 1911 his right arm became so seriously involved that it had to be amputated. Soon after this he retired from the Infirmary on a pension, and his health improved for a time, but afterwards cancer of the upper lip, due to the same cause, developed. His case was brought to the notice of the Carnegie Trust, which, in recognition of the self-sacrificing spirit he had shown, placed his name on the list of heroes and granted him a pension.

### Training for Public Health Positions.

Courses of lectures for Sanitary Officers, Meat and Food Inspectors, Health Visitors and Child Welfare Workers will be held at the Royal Sanitary Institute, beginning on January 28, February 8 and February 4 respectively. These training courses are of particular interest to students desiring to qualify for appointments on the staff of public health authorities. The training not only includes lectures, but practical demonstrations in the Museum of Sanitary Appliances, at Infant Consultations and Child Welfare Centres, visits to public abattoirs, fish markets, public works and other places of sanitary interest, and the use of a reference library, lending library and reading-room. The lectures are followed by the standard examinations of the Institute, which are recognised in all parts of the British Empire. Further particulars can be obtained from the Director of the Institute, 90 Buckingham Palace Road, S.W. 1.

### Empire Leprosy Campaign.

At a meeting of the General Committee of the British Empire Leprosy Relief Association, held recently, Sir Edward Gait, chairman of the Executive Committee, gave a report on the preliminary work accomplished since the Association was formed last summer. He stated that an opening meeting of the campaign would be held at the Mansion House on January 31, at 3 o'clock, and arrangements had been made for the broadcasting of a message by Lord Chelmsford and for some pictorial representation at the British Empire Exhibition. Sir Leonard Rogers, the hon. medical secretary, gave some details of the encouraging position for the application of the programme. He stated that the widespread extension of treatment now proved to be effective would be greatly assisted by the progress made in cheapening the cost. At present the treatment did not reach 5 per cent. of the 300,000 sufferers from leprosy in the Empire. A *questionnaire* drawn up by the Medical Committee had been circulated, through the kindness of the Foreign, Colonial and India Offices to every part of the Empire, and had been sent unofficially to every medical missionary in the King's Dominions. The results were to be collated and tabulated by card index. A sum of £250,000 is required to assist and co-ordinate the work of the existing agencies.

